# Application of Minimally Invasive Prosthodontics With an Esthetic 16-Unit Rehabilitation of the Dental Arches for a Patient With Anterior Dental Crowding and a Relatively Deep Bite: A Case Report

**DOI:** 10.7759/cureus.71692

**Published:** 2024-10-17

**Authors:** Osama M. Hajeer, Amal Sabri Hasan, Shaza M.A. Kanout, Mahmoud Khaled Nasani, Mohammad Y. Hajeer

**Affiliations:** 1 Department of Fixed Prosthodontics, Faculty of Dentistry, University of Aleppo, Aleppo, SYR; 2 Department of Fixed Prosthodontics, Faculty of Dentistry, University of Damascus, Damascus, SYR; 3 Department of Endodontics and Operative Dentistry, Faculty of Dentistry, University of Aleppo, Aleppo, SYR; 4 Department of Orthodontics, Faculty of Dentistry, University of Damascus, Damascus, SYR

**Keywords:** ceramic veneers, crowding of lower anterior teeth, crown preparation, crowns and bridges, deep bite, minimally invasive dentistry, minimally invasive prosthodontics, oral rehabilitation, stained teeth

## Abstract

Minimally invasive dentistry indicates an operative intervention to correct or manipulate a dental anomaly or lesion while focusing on preserving the original tissues as much as possible. Losing teeth or teeth loss is one of the most common problems patients have always suffered from due to different reasons, such as caries, trauma, or periodontal problems. In cases of multiple teeth loss, oral rehabilitation is usually indicated. Aesthetic reconstruction of a patient’s mouth with crowded teeth and a deep bite is challenging for prosthodontists. This case report shows an applicable conservative approach of minimally invasive dentistry in this situation while maintaining vertical dimension and centric occlusion.

## Introduction

Minimally invasive dentistry indicates an operative intervention to correct or manipulate a dental anomaly or lesion while focusing on preserving the original tissues as much as possible [1]. Losing teeth or teeth loss is one of the most common problems patients have always suffered from due to different reasons, such as caries, trauma, or periodontal problems. In the cases of multiple teeth loss, oral rehabilitation is usually indicated [2].

When oral rehabilitation is sought, patients usually visit dentists with many complaints, especially aesthetical ones, affecting their overall health, mental well-being, and psychosocial health status [3]. Therefore, oral rehabilitation has become important in re-designing a functional occlusion and providing an esthetically pleasing smile through several restorative and prosthetic procedures. [4] Such as traditional crowns and bridges that require a lot of preparation of the dental tissues, while the same result could be achieved using less invasive methods [1]. Therefore, the term ‘minimally invasive prosthodontics’ has emerged, in which conservative methods are usually adopted to restore the functional and esthetic properties of the tooth without harming the original tooth structure or tissues.

Crowding is one of the most repetitive malocclusion cases requiring clinical intervention. It happens in all ages, causing the teeth to overlap each other. Leading to a bad aesthetic look [5]. Deep bites are another common malocclusion in day-to-day dentistry practice; the vertical overlap increase between the maxillary and mandibular anterior teeth can be dealt with by several approaches, from orthodontics to surgery [6]. Although correcting deep bites by reorganizing the occlusion at an increased vertical dimension of occlusion (VDO) has been well documented, some complaints were found commonly [7]. The presented case report shows a treatment sequence provided for a patient with multiple aesthetic complaints employing the principles of ‘minimally invasive prosthodontics’ to achieve a reasonably satisfying result regarding esthetics and function. 

## Case presentation

Patient's chief complaint and clinical examination

**Figure 1:**
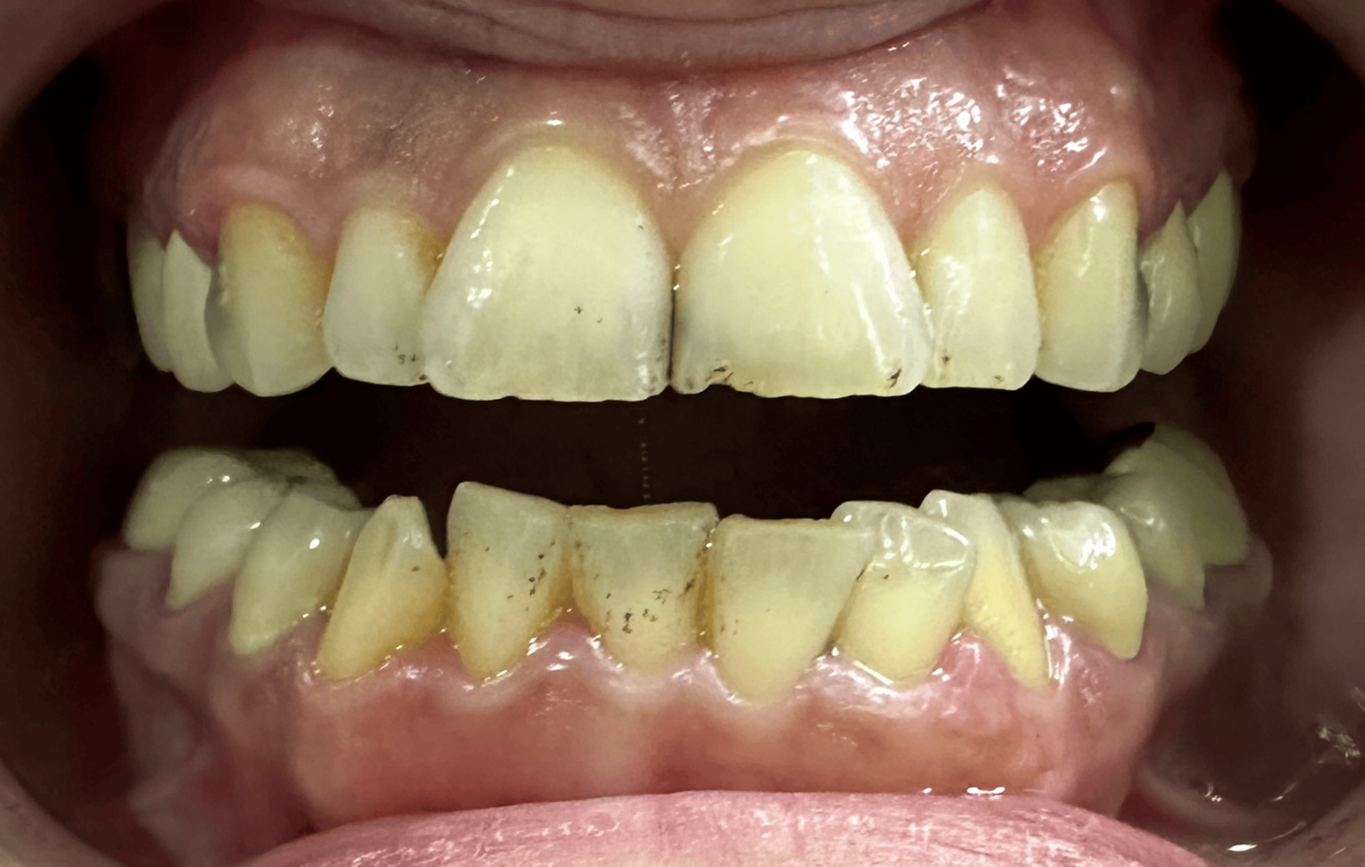
An intra-oral frontal photograph of the upper and dental arches with the mouth open.

**Figure 2:**
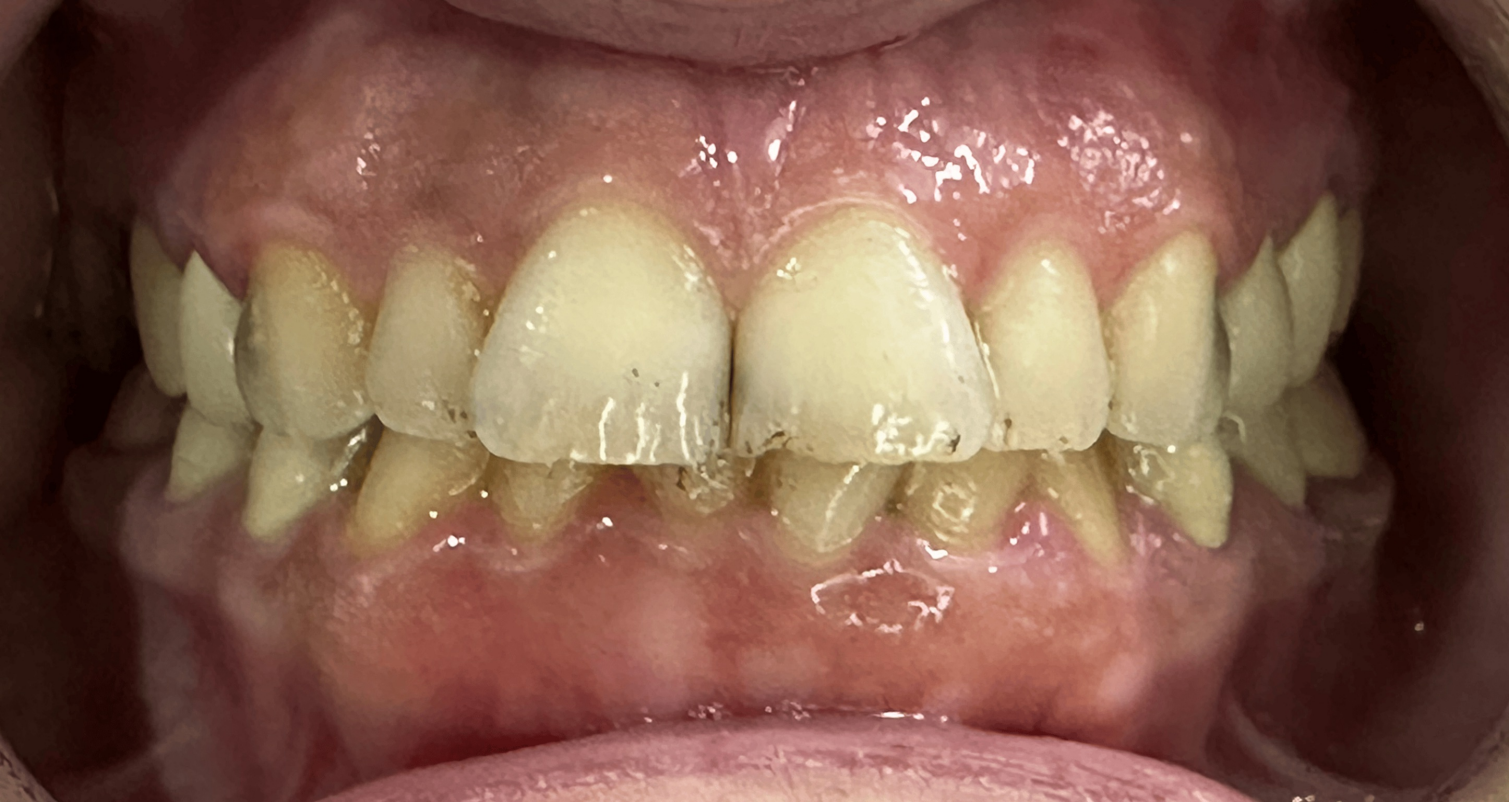
An intra-oral frontal photograph in centric occlusion. The bite was relatively deep.

A 43-year-old woman was referred to the Department of Fixed Prosthodontics at Aleppo University for oral rehabilitation. The clinical history of past dental interventions performed on her was taken, along with photographs that determined the mentioned interventions. Her chief complaint was the poor esthetic characteristics of her teeth, especially in the anterior region of both the upper and lower dental arches (Figures 1, 2).

No apparent caries were detected when the intraoral examination was performed.The oral hygiene was appropriate, with sufficient attached gingiva. Tooth size abnormalities were found, especially in the maxillary central incisors. In addition, smile disharmony and crowded mandibular teeth were also revealed, especially in the anterior region of the mandibular arch. The teeth were also tilted and rotated. No abrasions, attritions, erosions, or abfractions were detected. In addition, the patient's overall hygiene was relatively good. Teeth #1, #16, #17, #18, #31, and #32 were missing. Teeth #8 and #9 were tilted distobuccally and were of abnormal sizes, which caused lateral drifts to teeth #7 and #10, which were also palatally tilted, especially in tooth #7. Anterior teeth in the lower arch were crowded; #22 tilted lingually, #23 rotated clockwise, #24 tilted buccally, and #26 tilted lingually. The occlusal plane was harmonic. Static occlusal examination showed a deep bite. Maxillary lateral incisors were also tilted palatally, causing esthetic disturbances. The upper and lower midlines were not coincident, but the lower arch did not appear in the smiling view. The marginal gingival line was relatively even in both maxillary and mandibular arches. There was no surface loss in the teeth and the enamel was present and in adequate thicknesses (Figure 1). Deep caries in teeth #6 and #11 were detected radiographically. Teeth #4, #5, #12, and #13 had porcelain fused to full metal crowns, which poorly matched the color of the remaining teeth and did not have an appropriate contour. The teeth with abnormalities in sizes, shapes, and positions are shown in the smiling view.

The vertical dimension of occlusion was evaluated, 3 millimeters of freeway space was found, and it lay within the normal range [8]. No apparent dysfunctions, bad habits, or special diets. There were also no clenching symptoms or malfunctions in the masticatory system. The temporomandibular joint (TMJ)was normal and functional, with no abnormal deformities. There were no deviations in the maximum opening movements. Extraoral examination showed a high level of symmetry alongside esthetic proportions of the face with no factors limiting the movement of the patient's mouth. There was no loss or augmentation in the vertical proportions of the face. No abnormalities were found when palpating muscles and lymph nodes.

Radiographic examination

**Figure 3:**
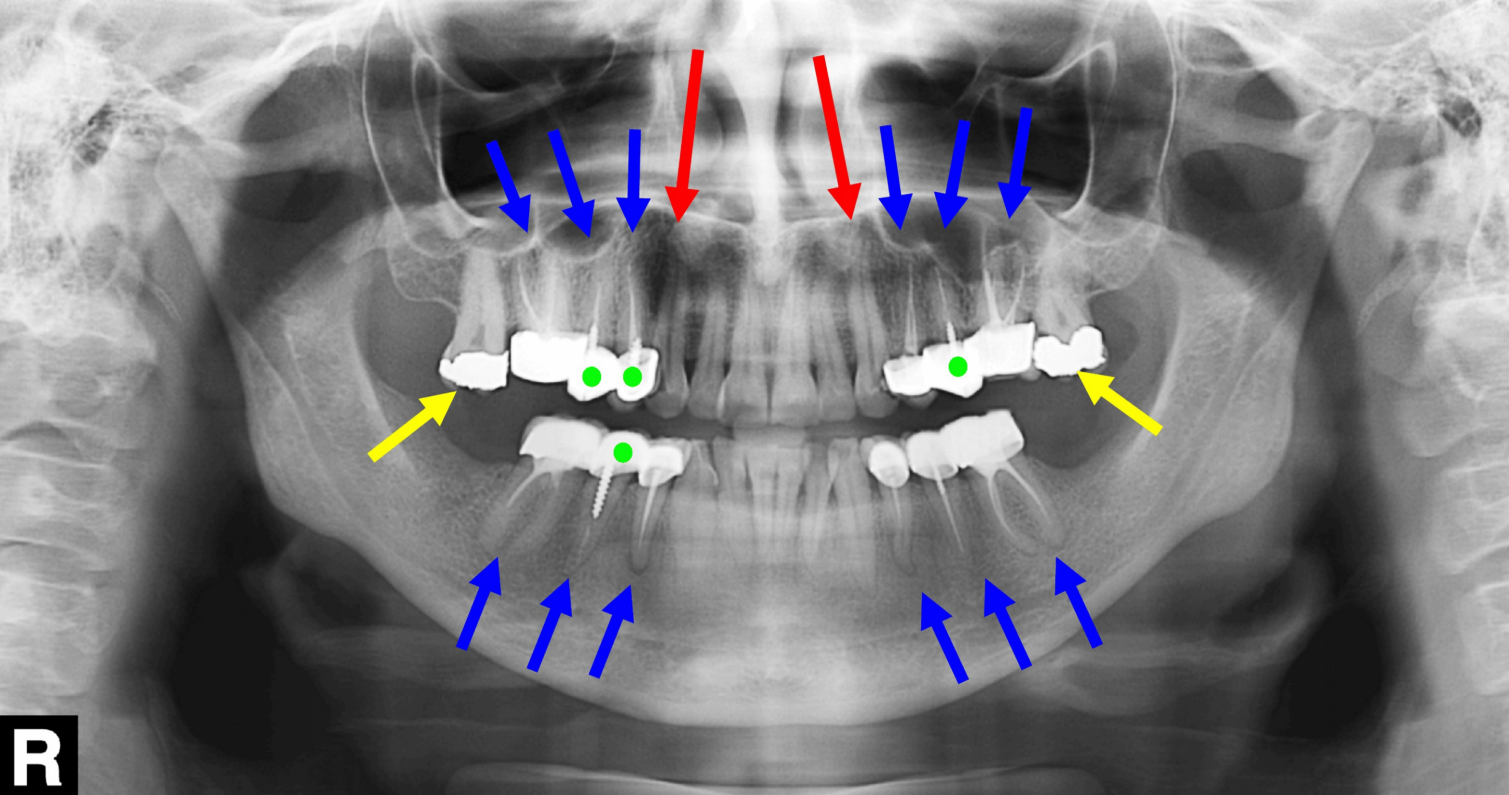
A panoramic radiograph of the dentition at the first visit. The blue arrows indicate the teeth that required root canal re-treatment. The red arrows indicate the teeth that required root canal treatment (RCT). The yellow arrows indicate two teeth with amalgam fillings. The green dots indicate the teeth with screw-retained metal posts.

The crown-root ratio in teeth was good in the radiographic periapical view. Teeth #3, #4, #5, #12, #13, #14, #19, #20, #21, #28, #29, and #30 had unacceptable Root Canal Treatments (RCTs). Teeth #6 and #11 needed RCT. Teeth #2 and #15 had an amalgam filling, which was neither aesthetic nor durable. Teeth #4 #5 #12 #13 #29 had non-durable metal screw posts (Figure 3).

Treatment planning, smile evaluation, and esthetic planning

As for the diagnostic stage, primary alginate impressions (Hygedent, Beijing Haijiya Medical Equipment Co., Beijing, China) were made and poured by plaster (Moldano Stone, Bayer Co., Whippany, United States). Centric occlusion was recorded using bite registration silicone (Futar D, Kettenbach GmbH & Co., Eschenburg, Germany). Vertical Dimension of Occlusion (VDO) was evaluated in the centric occlusion (CO) as the patient had enough space for aesthetic rehabilitation without occlusal interferences. A 10-maxillary-unit from the second left premolar to the second right premolar and a 10-unit esthetic rehabilitation on the mandibular side, too, was suggested to improve the patient's smile esthetics.

Treatment options 

**Figure 4:**
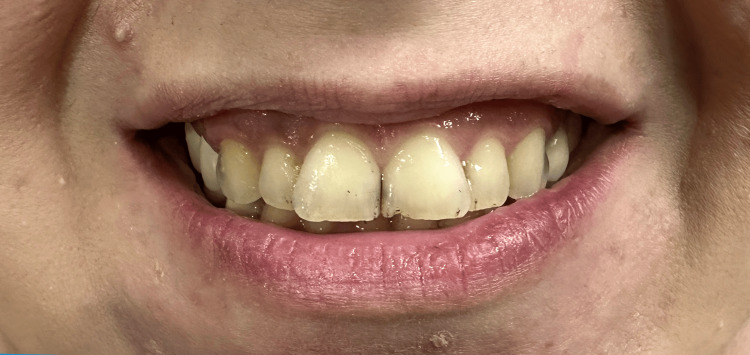
An extraoral frontal smile view. Only upper anterior teeth are seen during this posed smile.

This rehabilitation procedure could be achieved by applying traditional crowns on intact teeth and replacing old crowns and bridges with normal ones. This solution was proposed to the patient, and the advantages and disadvantages of conventional crowns and bridges were also explained. She was informed that this traditional method would be invasive and waste too much tooth structure. Thus, applying laminate veneers was a more reasonable option because only the anterior ten ((from the right second premolar to the left second one in the upper arch)) was seen in the patient’s smile (Figure 4). The patient showed an apparent desire only to reconstruct the upper arch. Then, the patient accepted mandibular anterior six to undergo rehabilitation as they were heavily tilted, rotated, and crowded. She proclaimed that she would continue this procedure for the lower posterior region once she gets some rest after the first stage is done. The patient showed concerns about how much of the tooth structure would remain. In addition, applying the most efficient aesthetic treatment and the least invasive to the patient teeth was needed. Thus, minimal preparations were indicated.

Treatment sequence

Preparatory Stage

Root canal treatments (RCTs) were done, and old Porcelain-Fused-to-Metal (PFM) crowns on teeth #4 #5 #12 #13 were removed, and the screw posts in teeth #4 #5 #13 were replaced with fiber posts. RCT on teeth #6 and #11 was done due to deep caries that reached the pulp tissue

Restorative Stage

Emax fused-to-zirconia crowns were applied on teeth #4 #5 #12 #13. Then, three-quarters of Emax crowns were used on teeth #6 and #11. At the same time, porcelain laminate veneers with palatal overlap design were applied for maxillary medial and lateral incisors teeth (i.e., #7, #8, #9, and #10).

For the lower arch’s anterior six, it was challenging whether we could maintain the preparation within the enamel region for optimal resin cement adhesion [9]. After applying the wax and preparing through it, it was decided that laminate veneers could be used on teeth #22, #23, #24, #25, #26, and #27 to restore aesthetics and fix crowding due to sufficient remaining enamel thickness. Moreover, retention and stability were thought to be maximized in these teeth using the selective etch method on dentine using a universal bond [10]. Thus the probability of having to undergo an endodontic treatment if post-prosthodontic complications occur would be minimized [11], plus, no natural tooth tissues would be wasted in the procedure.

Teeth preparation and smile design protocol

**Figure 5:**
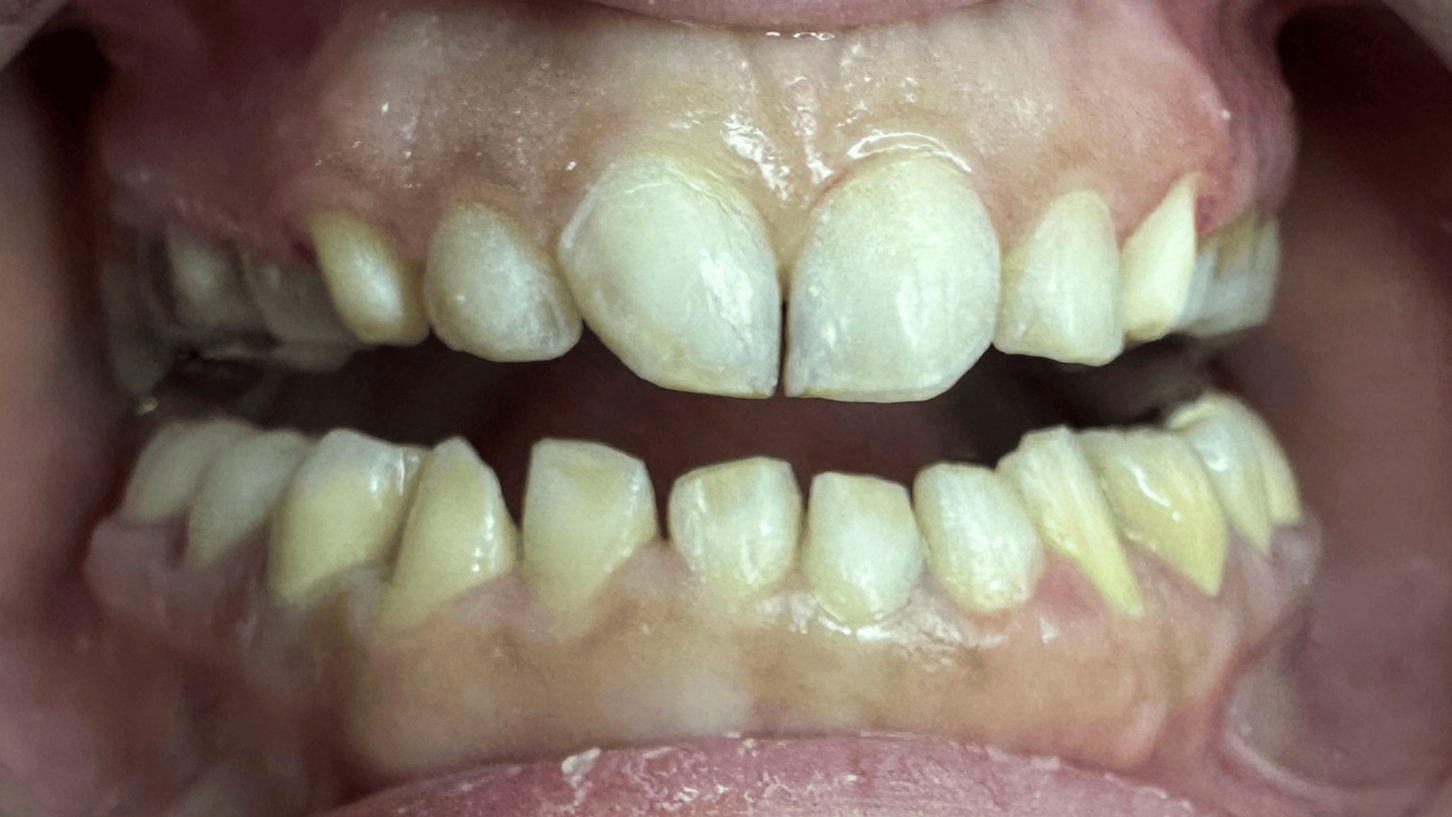
An intraoral frontal photograph showing the prepared teeth.

The protocol of preparation was the following: first, a supra-gingival finish line (0.5 mm supra-gingivally) was employed [12], and for the thicknesses of this finish line, circumferential radial shoulder finish lines for the crowns on teeth #4, #5, #12, and #13 were done. At the same time, light chamfers (approx. 0.3 mm) were adopted for veneers and 3/4 Emax crowns on canines with incisal overlaps on the prepared teeth for laminates, which are known to have the most predictable outcome (Figure 5) [13].

**Figure 6:**
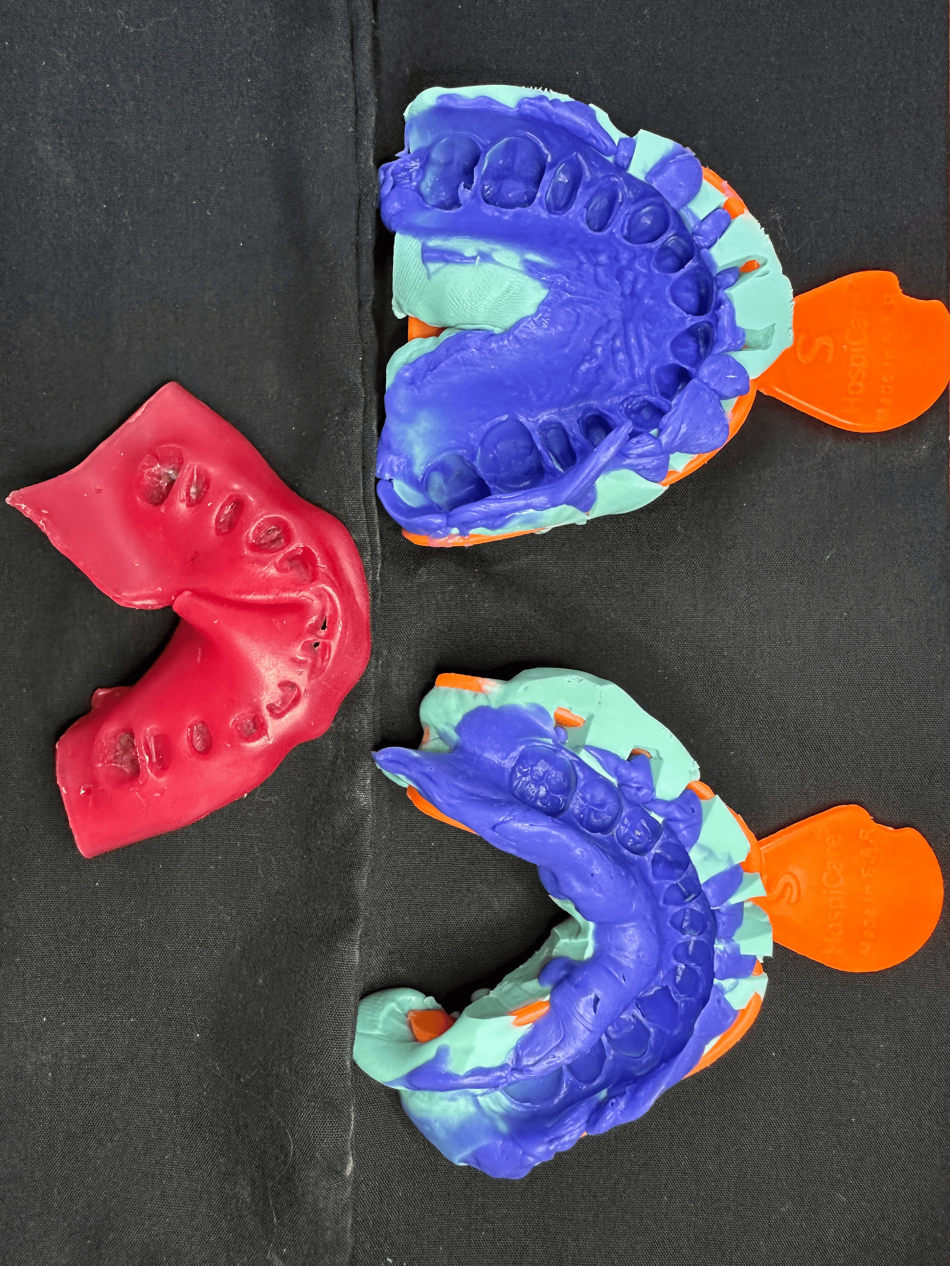
Final maxillary and mandibular silicone impressions with a wax bite registration.

Final impressions were made using a two-stage putty wash technique (Zeta-plus, Zhermack, Ferrara, Italy; Figure 6). Exocad® software ((Exocad Co., Darmstadt, Germany)) was used to design the zirconia cores, and frameworks were milled. IPS e.max® ZirCAD (Ivoclar Vivadent, Liechtenstein) for Emax fused to zirconia crowns and IPS e.max cad (Ivoclar Vivadent, Liechtenstein) for veneers and 3/4 crowns. Then, the cores were intra-oral-checked, marginal integrity was tested using radiographs, and Emax was applied to the frameworks. The porcelain-fused prosthesis was tested on esthetics, and the margins were confirmed. Eventually, glazing was done after the final application of the crowns and veneers.

Bonding of the veneers, cementation of the crowns, and the outcome

**Figure 7:**
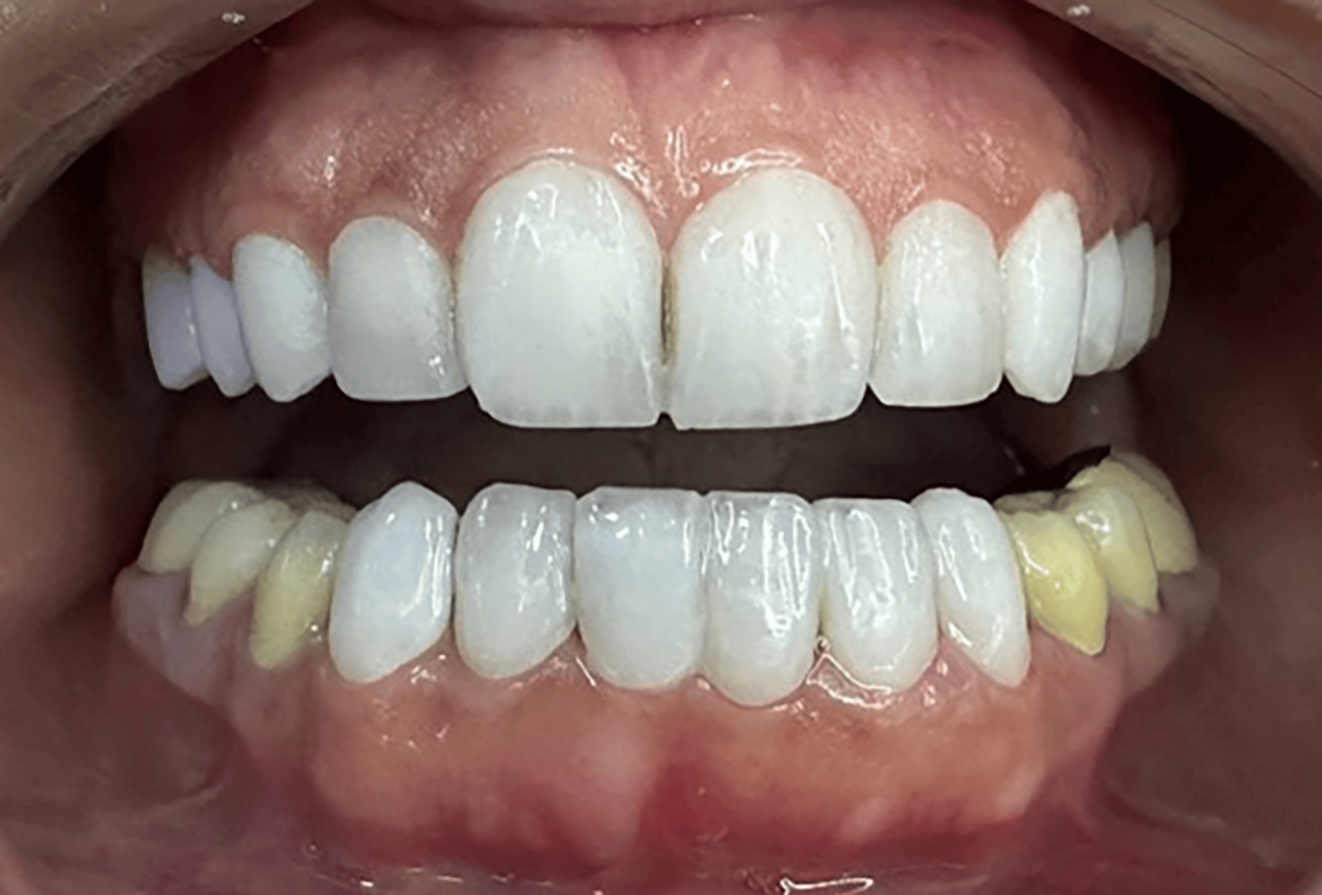
An intraoral frontal photograph with the mouth open. This photograph shows the final outcome following the bonding of the upper four veneers, two three-quarter crowns, and two crowns, and the lower six veneers.

**Figure 8:**
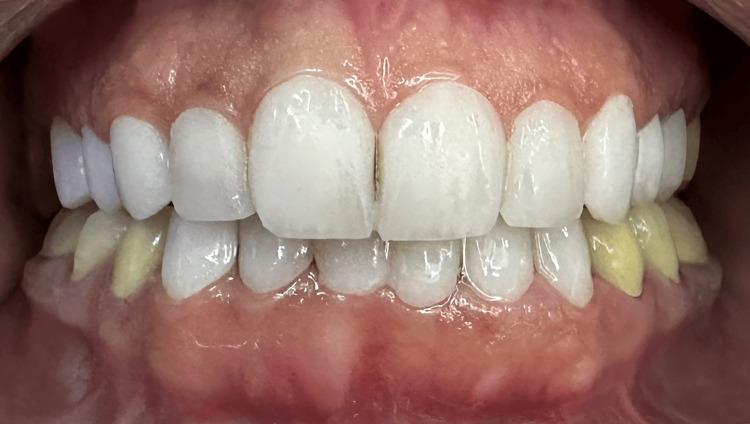
An intraoral frontal photograph of the dental arches in centric occlusion.

In the delivery session, veneers and three-quarters crown adhesion was performed using Choice 2 Veneer Cement (BISCO Dental, USA). The full crowns were cemented using glass ionomer cement (GIC)(Fuji II, GC Dental Corp, Tokyo, Japan; Figures 7, 8).

**Figure 9:**
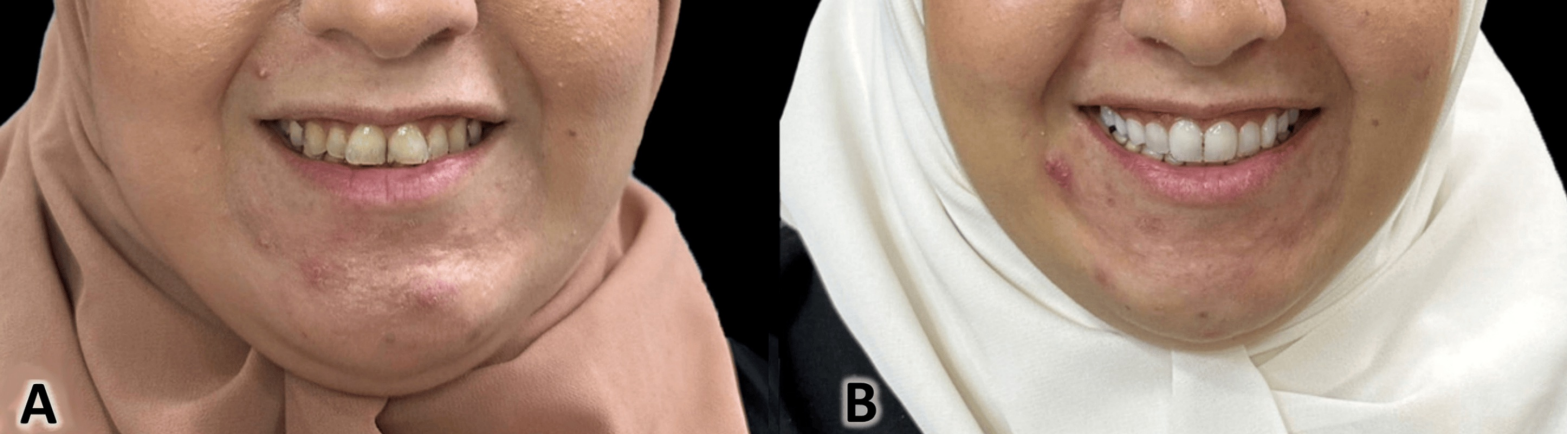
Extra-oral photographs of the lower part of the face before (A) and after treatment (B). The patient was in a posed smile position.

After the delivery of restorations, oral hygiene instructions using dental brushes and floss threaders were explained to the patient, and follow-ups were set for 1, 6, and 12 months. In the first and second follow-ups (up to the date of the report), the patient was significantly happy and satisfied with the results, especially ones related to the esthetic part (Figure 9).

**Figure 10:**
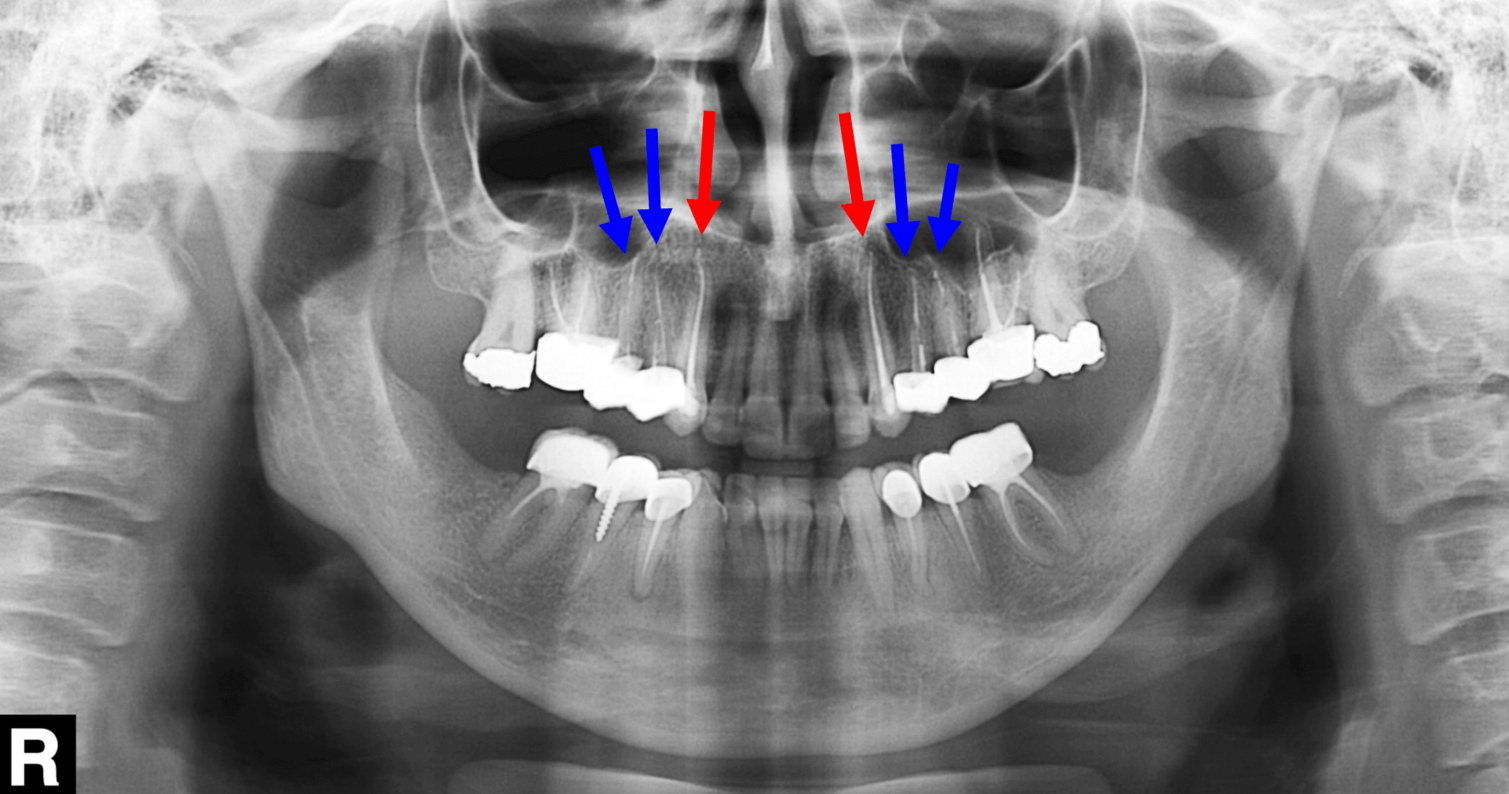
A panoramic radiograph of the dental arches immediately post-treatment. The red arrows indicate the upper canines following root canal treatment, whereas the blue arrows indicate the teeth that underwent root canal re-treatment.

**Figure 11:**
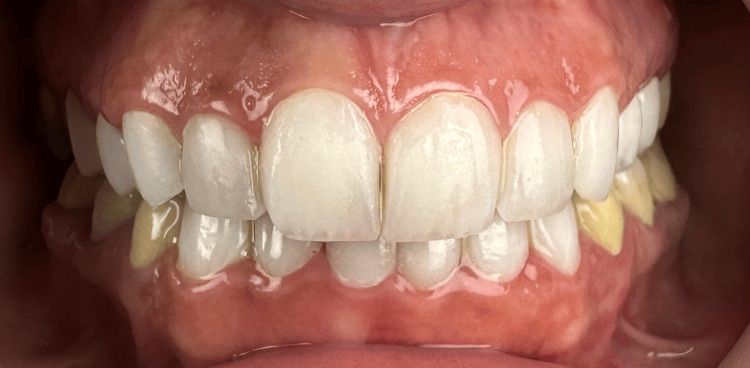
An intraoral fontal photograph of the dental arches in centric occlusion taken at six months post-treatment.

**Figure 12:**
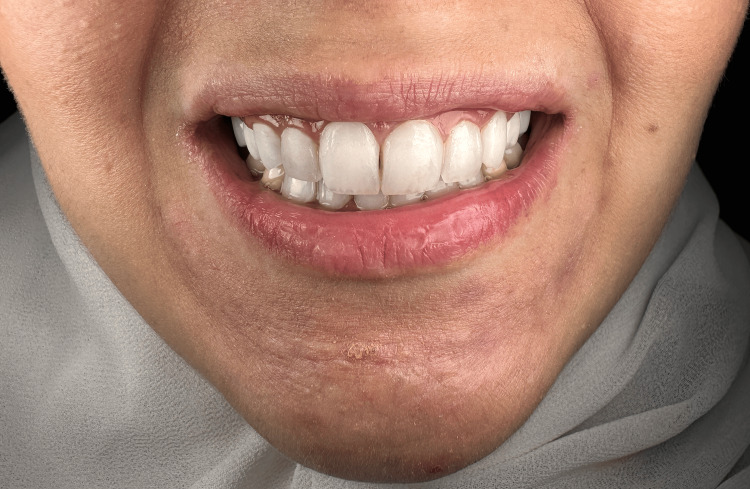
An extraoral frontal photograph of the face with the patient in posed smile taken at six months post-treatment.

On the other part, the functional one, there were no complaints about mastication problems or temporomandibular joint complications. There were also no tensions in the mastication muscles as the prosthetic procedure remained in the safe zone, i.e., the centric occlusion. There were also no periodontal complications, the marginal gingival line was stable, and no recession nor inflammation was found due to the 0.5 supragingival preparation being used in the procedure and the proper placement of the prosthesis with all excess cement removed immediately after adhesion. The panoramic radiograph taken immediately post-treatment showed no signs of untoward effects (Figure 10). All the restorations were structurally stable, with no apparent cracks, attritions, or discolorations at six months post-treatment (Figures 11, 12).

## Discussion

The current case report focused on the minimally invasive interventions that could be performed when a patient with esthetic complaints seeks prosthodontic treatment. The report aims to show an attempt to achieve esthetic oral rehabilitation by employing conservative methods with no periodontal surgery. Only scaling and root planning were done on the periodontal side.

The deep bite shown in the patient’s frontal view in centric occlusion before the treatment photo was eased by re-defining teeth lengths while maintaining and even improving the esthetic aspects and by sticking to the 1.6-1-0.6 ratio, which is the ratio of the widths of central incisor-lateral incisor-canine, when widths are measured from the mesial contact point to the distal-most visible point in the frontal view, or what is called ‘the golden ratio’. Taking into consideration the following smile design principles: The incisors’ width-to-length ratios are 75-80% - oral and facial midlines should be symmetric - gingival margin line heights are adequate - zenith points in their optimum mesiodistal positions - horizontal planes symmetry whether intraorally or extraorally [11]. By doing that, the esthetic rehabilitation was achieved without changing the vertical or centric occlusion dimension. Otherwise, it would have been obligating to increase the VDO and rely on the Centric Relation (CR) [14].

The minimally invasive approach (using only veneers and three-quarters crowns) meant only minimized preparation was performed, and strong ceramics (crystalline-dominated Emax) that could withstand high forces even when small thicknesses were used [15] all helped maintain the VDO.

One of the challenges, in this case, was how to use crowded teeth with only minimal preparations so that enamel thicknesses that are mandatory for the adhesive process could be maintained to achieve the minimally invasive prospect efficiently. The anterior deep bite issue was also a challenge. It is mentioned in some cases, especially cases with implants, that when increased, the vertical dimension of occlusion does allow for enough space, one that can be used to manage an anterior overbite. The changes in the VDO may be adaptable in the TMJ, the periodontium, and the occlusion morphology [16]. On the contrary, some studies have suggested that increasing the vertical dimension of occlusion could harm the patients, be hard to adapt to, and be a disturbance to the physiology of the muscles and the temporomandibular joint [17].

Six laminate veneers on the mandibular anterior were manufactured using pure Emax, which had superior aesthetics [18]. In premolars, using zirconia as a core was obligatory because the teeth were previously treated with a crown. This gave high mechanical durability since zirconia is known for its high wear resistance [19]. However, Emax was also fused to these teeth to make esthetics as high as possible.

Another thing to mention is that the upper and lower midlines in the final result were not coincident, yet the upper midline was in line with the facial midline, which is more important aesthetically. The lower midline, also, as mentioned in the presentation, was not apparent in the view of the frontal smile. Thus, it was not important to fix this problem; it was also impossible to do so using minimally invasive methods. A study mentioned that a discrepancy between the upper and lower midlines is only one-fourth of the population [20]. The maxillary and mandibular midline discrepancy occurs naturally to one side of the face, so if mandibular incisors and midline are less apparent due to the deep bite and a refusal of orthodontic treatment is also present while relying on veneers as a treatment, this discrepancy cannot be avoided [20].

The patient's main complaint was completely solved at the end of treatment. She was reassured and happy with the aesthetic qualities of her new smile. After six months, the prosthesis had no symptoms of fracture or wear and was all smooth and esthetic. The reason may be attributed to the preservation of the VDO and the centric occlusion with no apparent unfavorable habits or parafunctional activities of the masticatory muscles, which resulted in intact, healthy, stable, and aesthetic restoration.

## Conclusions

Aesthetic rehabilitation demands high cooperation between multiple dentistry disciplines to acquire favorable esthetic results while maintaining the mouth's functionality as a whole. It is essential to verify that, in this case, the sequence of procedures was performed employing a minimally invasive approach. The treatment maintained the current centric occlusion and vertical dimension to meet all biological and mechanical demands and prevent probable complications that may arise due to changes in the mentioned determinants. Therefore, the outcome of the provided treatment was a better smile with healthy dentition and functional occlusion.
